# Supplementary material for: Identification of photoperiod-induced specific miRNAs in the adrenal glands of Sunite sheep (Ovis aries)
Source: Front Vet Sci. 2022 Jul 22;9:888207. doi: 10.3389/fvets.2022.888207 (PMC9354845; doi:10.3389/fvets.2022.888207)
Supplement: Supplementary file 1 [file Data_Sheet_1.ZIP › Supplementary file/miRDeep2 file.zip!/miRDeep2 file/Know_Structure/SP42A2/oar-mir-668.pdf]

miRBase precursor : oar-mir-668
Total read count : 52
oar-miR-668-5p read count : 3
oar-miR-668-3p read count : 49
remaining reads : 0

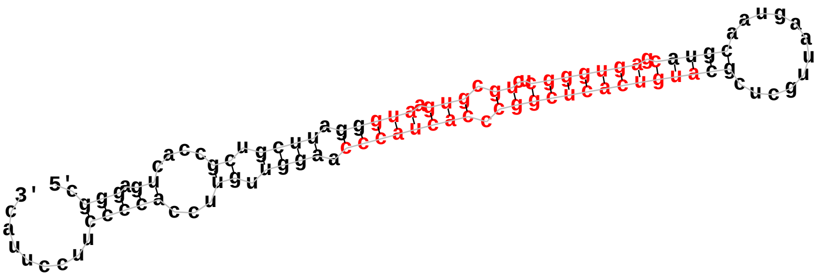

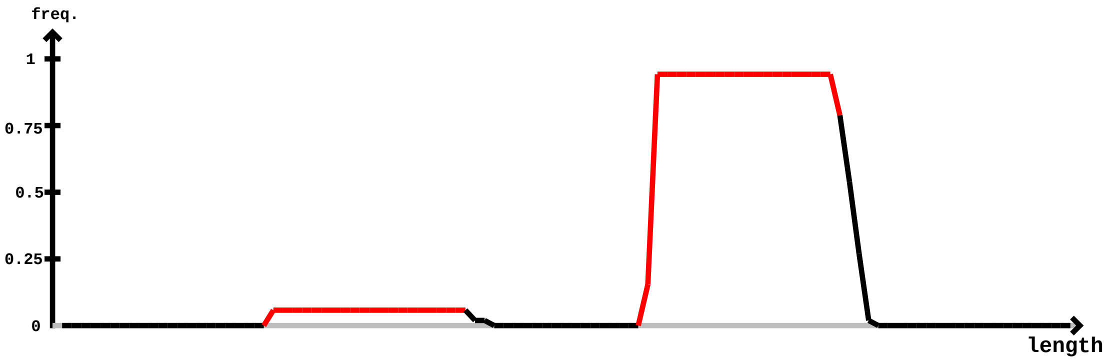


```
                                                              oar-miR-668-3p
                        oar-miR-668-5p
5'- cgggagucaccgcugcuuaggguaagugcgucucgggugagcaugcaaugaauugcucgcaugucacucggcccacuacccaagguuguuccaccccuuccuuac   -3'    exp
    .(((.((....((.((((.(((((.(((.((..(((((((.(((((............))))))))))))))).)))))))).)))).))...)))))........    reads     mm       sample
    .....................guaagugcgucucgggugagc...............................................................    2         0        seq
    .....................guaagugcgucucgggugagcau.............................................................    1         0        seq
    ............................................................augucacucggcccacuacc.........................    4         0        seq
    ............................................................augucacucggcccacuaccc........................    2         0        seq
    ............................................................augucacucggcccacuaccca.......................    2         0        seq
    .............................................................ugucacucggcccacuacc.........................    4         0        seq
    .............................................................ugucacucggcccacuaccc........................    11        0        seq
    .............................................................ugucacucggcccacuaccca.......................    12        0        seq
    .............................................................ugucacucggcccacuacccaa......................    13        0        seq
    .............................................................ugucacucggcccacuacccaag.....................    1         0        seq
```
